# Pearson’s Appointment Book

**Published:** 1883-11

**Authors:** 


### PEARSON'S APPOINTMENT BOOK.

We have received from the publisher, J. L. Brewster, Jr., of Kansas City, a very neat and convenient appointment book, intended to be carried in the vest pocket, yet large enough for the recording of all the appointments of a year, with the operation to be performed. Each day has eight lines appropriated to it, and as the dates are not filled out it is good for any year. It may be procured at the Kansas City dental depot.

